# Supplementary material for: Pulsed ultrasound promotes secretion of anti-inflammatory extracellular vesicles from skeletal myotubes via elevation of intracellular calcium level
Source: eLife. 2023 Dec 6;12:RP89512. doi: 10.7554/eLife.89512 (PMC10699803; doi:10.7554/eLife.89512)
Supplement: Supplementary file 1. — miRNA-sequencing analysis in extracellular vesicles from US-treated/untreated C2C12 myotubes was conducted. [file elife-89512-supp1.docx]

**Supplemental material 1-1**

List of specific miRNAs in the control group

| miRNA |
| --- |
| miR-7043-3p |
| miR-3087-3p |
| miR-8112 |
| miR-485-3p |
| miR-185-3p |
| miR-7648-3p |
| let-7i-3p |
| miR-1960 |
| miR-3061-3p |
| miR-3061-5p |
| miR-494-3p |
| miR-1934-5p |
| miR-1941-5p |
| miR-6914-3p |
| miR-6956-3p |
| miR-431-3p |
| miR-205-5p |
| miR-187-5p |
| miR-6948-3p |
| miR-6966-3p |
| miR-3473h-5p |
| miR-323-3p |
| miR-8103 |
| miR-328-5p |
| miR-382-5p |
| miR-504-5p |
| miR-8105 |
| miR-23a-5p |
| miR-6928-3p |

**Supplemental material 1-2**

List of specific miRNAs in the ultrasound group

| miRNA |
| --- |
| miR-33-5p |
| miR-449a-5p |
| miR-467e-5p |
| miR-1929-5p |
| miR-1963 |
| miR-1966-5p |
| miR-25-5p |
| miR-196b-3p |
| miR-700-5p |
| miR-8118 |
| miR-12193-5p |
| miR-130b-3p |
| miR-7660-3p |
| miR-33-3p |
| miR-146b-3p |
| miR-675-5p |
| miR-3095-5p |
| let-7g-3p |
| miR-138-5p |
| miR-450b-5p |
| miR-324-3p |
| miR-743b-5p |
| miR-669a-5p |
| miR-669a-5p |
| miR-669a-5p |
| miR-669a-5p |
| miR-669a-5p |
| miR-669a-5p |
| miR-669a-5p |
| miR-669a-5p |
| miR-669a-5p |
| miR-669a-5p |
| miR-669a-5p |
| miR-669a-5p |
| miR-669p-5p |
| miR-669p-5p |
| miR-130a-5p |
| miR-6994-3p |
| miR-124-5p |
| let-7j |
| miR-542-5p |
| miR-7653-5p |
| miR-6901-5p |
| miR-452-3p |
| miR-7065-3p |
| miR-147-5p |
| miR-212-3p |
| miR-138-2-3p |
| miR-31-3p |
| miR-129-1-3p |
| miR-1930-5p |
| miR-3082-3p |
| miR-345-5p |
| miR-10a-3p |
| miR-744-3p |
| miR-1306-5p |
| miR-6945-3p |
| miR-153-5p |
| miR-324-5p |
| miR-298-3p |
| miR-7083-5p |
| miR-181a-1-3p |
| miR-7685-3p |
| miR-135b-5p |
| miR-150-5p |
| miR-1955-5p |
| miR-485-5p |
| miR-6948-5p |
| miR-465a-5p |
| miR-301a-5p |
| miR-344c-3p |
| miR-7021-5p |
| miR-223-5p |
| miR-3094-3p |
| miR-488-3p |
| miR-370-5p |
| miR-677-5p |
| miR-5132-5p |
| miR-28c |
| miR-200a-5p |
| miR-3060-3p |
