# Supplementary material for: Pulsed ultrasound promotes secretion of anti-inflammatory extracellular vesicles from skeletal myotubes via elevation of intracellular calcium level
Source: eLife. 2023 Dec 6;12:RP89512. doi: 10.7554/eLife.89512 (PMC10699803; doi:10.7554/eLife.89512)
Supplement: Supplementary file 3. [file elife-89512-supp3.docx]

**Supplemental material 3**

Sequences for qPCR primers.

| Primer | Sequences |
| --- | --- |
| *Gapdh*-Forward | 5'-CCAATGTGTCCGTCGTGGATCT-3' |
| *Gapdh*-Reverse | 5'-GTTGAAGTCGCAGGAGACAACC-3' |
| *Il-1b*-Forward | 5'-GCCTTGGGCCTCAAAGGAAAGAA-3' |
| *Il-1b*-Reverse | 5'-ATTGCTTGGGATCCACACTCTCC-3' |
| *Il-6*-Forward | 5'-ACAAAGCCAGAGTCCTTCAGAGA-3' |
| *Il-6*-Reverse | 5'-TTGGATGGTCTTGGTCCTTAGCC-3' |
